# Polysaccharides from *Eucommia ulmoides* Oliv. Leaves Alleviate Acute Alcoholic Liver Injury by Modulating the Microbiota–Gut–Liver Axis in Mice

**DOI:** 10.3390/foods13071089

**Published:** 2024-04-02

**Authors:** Yingzhi Li, Huimei Wang, Xueping Leng, Jiaming Gao, Chang Li, Danfei Huang

**Affiliations:** 1 State Key Laboratory of Food Science and Resources, China-Canada Joint Lab of Food Science and Technology (Nanchang), Key Laboratory of Bioactive Polysaccharides of Jiangxi Province, Nanchang University, 235 Nanjing East Road, Nanchang 330047, China; 15025126433@163.com (Y.L.); whm20220602@163.com (H.W.); lengxueping12@163.com (X.L.); gaojiaming8712@163.com (J.G.); lichang@ncu.edu.cn (C.L.); 2 International Institute of Food Innovation Co., Ltd., Nanchang 330200, China

**Keywords:** polysaccharides, acute alcoholism, liver injury, inflammation, antioxidant, microbiota–gut–liver axis

## Abstract

The interplay among gut microbiota, intestines, and liver is crucial in preventing acute alcoholic liver injury. In this study, the hepatoprotective potential of polysaccharides from *Eucommia ulmoides* Oliv. leaves (EULP) on acute alcoholic liver injury in Kunming male mice was investigated. The structural features suggested that the EULP appeared as a heterogeneous mixture of polysaccharides with a molecular weight of 186132 Da. A 14-day pretreatment of EULP ameliorated acute alcoholic-induced hepatic inflam mation (TNF-α, IL-6, and IL-10), oxidative stress (GSH, SOD, and T-AOC), and liver damage (ALT and AST) via enhancing intestinal barrier (Occludin, Claudin 1, and ZO-1) and modulating microbiome, which subsequently inhibiting endotoxemia and balancing the homeostasis of the gut–liver axis. EULP restored the composition of intestinal flora with an increase in the relative abundance of *Lactobacillaceae* and a decrease in *Lachnospiraceae* and *Verrucomicrobiaceae*. Notably, prolonged EULP pretreatment (14 days) but no single gavage of EULP achieved excellent hepatoprotection. These findings endorsed the potential of EULP as a functional food for mitigating acute alcoholic-induce d liver damage, attributed to its anti-inflammatory, antioxidant, and prebiotic properties facilitated by the microbiota–gut–liver axis.

## 1. Introduction

With the development of China’s economy, China’s per capita commercial alcohol production has increased more than 50 times since 1952 [1]. Current research has demonstrated that excessive drinking can increase the incidence of hypertension, diabetes, and hepatitis and interfere with the body’s metabolism and the therapeutic effects of certain medications. In addition, the rates of serious injury, accidental death, loss of income, overuse of health-care resources and interruption of family life due to alcoholism are also increasing [2,3,4,5]. Excessive alcohol consumption increases mortality rates by increasing the risk of liver injury, emphasizing the need for effective treatments. However, there are still no effective treatments for acute alcoholic liver damage.

Many studies have pointed to potential pathogenic mechanisms associated with alcohol-induced liver injury: oxidative stress in the gut–liver axis, inflammatory mediator injury, and nutritional imbalance. Moreover, there are studies that show that an imbalance in intestinal flora is a pivotal factor in the initiation and progression of alcoholic liver injury [6,7]. Recently, polysaccharides have garnered a lot of attention in alcohol liver injury due to their varied actions, such as anti-inflammation, antioxidant, anti-tumor, and enhancement of immune activity [8,9,10,11,12,13,14,15], as well as the ability to regulate the gut microbiota, thereby exerting positive efforts on host health [16,17].

*Eucommia ulmoides* Oliv. (also called Du-Zhong in Chinese), the bark of the plant *Eucommiaceae*, is a traditional Chinese medicinal plant with a medicinal use history dating back over 2000 years. Numerous pharmacological studies have demonstrated that *E. ulmoides* Oliv. has many pharmacological activities such as lowering blood pressure, enhancing immune activity, and lowering blood sugar [10,18,19]. The leaves of *E. ulmoides* Oliv., which have been proven to have similar compounds and replace the bark of *E. ulmoides* Oliv. to some extent, were included in the Chinese Pharmacopoeia in 2005, as well as the list of edible Chinese herbal medicines in 2020. Du-zhong tea, derived from the aqueous extract of *E. ulmoides* Oliv. leaves, is widely used and recognized as a functional health supplement for weight loss [20] and antioxidants [21]. It has also been widely used as a folk treatment in South China to effectively relieve the symptoms of alcoholism. Several research have demonstrated its anti-inflammatory [22], antioxidant [9,21], hypoglycemic [23,24], renal-protecting [25], and intestinal health-promoting properties [26,27]. Polysaccharides, which are the main active component of *E. ulmoides* Oliv. leaves, were chosen as the target of this study. We investigated the hepatoprotective potential of *E. ulmoides* Oliv. leaves polysaccharides (EULP) from the perspective of the gut–liver axis. This issue focused on the preventive effect of EULP on liver injury caused by acute alcoholism, to provide a preliminary exploratory approach for solving the scientific challenge of botanical natural components alleviating acute alcohol toxicity.

## 2. Materials and Methods

### 2.1. Extraction and Separation of Polysaccharides from E. ulmoides Oliv. Leaves (EULP)

*E. ulmoides* Oliv. leaves were purchased from Suining County, Shaoyang City, Hunan Province. The appropriate amount of fresh leaf was dried, crushed, and passed through a sieve with a mesh size of 60. We added 5 times the volume of petroleum ether (Xilong Scientific, Guangzhou, China) to the powdered leaves and sonicated at 30 °C for 40 min to remove gum and lipids. After filtering and drying, the filter residue was combined with 5 times its volume of 80% ethanol (Xilong Scientific, Guangzhou, China), and the reflux was carried out twice at 80 °C for 2 h to remove small molecules of monosaccharides, disaccharides, oligosaccharides, and other substances. The processed powder was weighed precisely and extracted twice at 100 °C for 2 h with 20 times the volume of distilled water. The solution was concentrated, 1% papain (Beijing Solarbio Science & Technology company, Beijing, China) was added, and it was heated in a water bath with stirring at 60 °C for 1 h. The liquid was then heated at 100 °C for 15 min before being centrifuged at 5000 rpm for 5 min. Following protein removal, the concentrate was put into a dialysis bag (MW 3500 Da, Beijing Solarbio Science & Technology company, Beijing, China) and dialyzed by flowing water for 72 h. After dialysis, the extract was then treated with anhydrous ethanol to a final concentration of 80% (*v*/*v*) for 12 h at 4 °C to precipitate polysaccharides. The precipitates were lyophilized after centrifugation at 4000 rpm for 15 min. The lyophilized powder was EULP [10,28,29].

### 2.2. Characterization of EULP

#### 2.2.1. Physicochemical Property

Total sugar was measured using the phenol sulfuric acid method by a UV–vis spectrophotometer (Beijing General Instrument Co. Ltd., Beijing, China). The protein content was determined using the Bradford kit (Beyotime Biotechnology, Shanghai, China) with a microplate reader (Varioskan Flash, Thermo Fisher Scientific, Waltham, MA, USA). 

#### 2.2.2. Molecular Weight Distribution Analysis

The molecular weight (Mw) analysis was determined using gel permeation chromatography (GPC) on Waters E2695 MEH performance liquid chromatography (Thermo Fisher Scientific, USA). An Ultrahydrogel TM protective column (6 mm × 40 mm) and an Ultrahydrogel TM linear column (7.8 mm × 300 mm) were connected in series to equip the GPC system. Detection was performed on a differential refractometer (RID, 2414) coupled with a UV detector (VWD, 2489). The performance conditions were as follows: injection volume 150 μL (0.6 mg/mL, *w*/*v*), column temperature 35 °C, and the eluent was sodium chloride (0.02%, pH 6.0) at a flow rate of 0.6 mL/min. A calibration curve was established using the dextran series [30,31].

#### 2.2.3. Monosaccharide Composition Analysis

An amount of 5 mg of EULP powder was combined with 0.5 mL of 12 M sulfuric acid and cooled in an ice bath for 30 min. Subsequently, 2.5 mL of deionized water was added, and the mixture was heated in an oil bath at 50 °C for 120 min. After cooling to room temperature, 1 mL of sample was taken out, and the sample was adjusted to 5 mL with ultrapure water. The mixed liquid was filtered through a 0.22 μm membrane and analyzed by using an Agilent 1260 MEH performance liquid chromatograph (HPAEC, Agilent Technologies, Santa Clara, CA, USA). The analysis was performed using CarboPac TM PA20 protective column (3 mm × 150 mm) in series with CarboPac TM PA20 protective column (3 mm × 30 mm) as follows: 30 °C of column temperature, 10 μL of injection volume, a mixture of NaOH solution (250 nm/L) and ultra-pure water as mobile phase, 0.5 mL/min of flow rate. Mixed monosaccharide standards with different concentrations were prepared using ten monosaccharide standards including glucose, xylose, fructose, arabinose, galactose, fucose, rhamnose, mannose, galacturonic acid, and glucuronic acid. We then identified and quantified various sugars by comparing the retention time and peak area with those of monosaccharide standards.

### 2.3. Animal Modeling

Four-week-old specific pathogen-free (SPF) Kunming male mice (24–32 g) were purchased from Zhejiang Weitong Lihua Experimental Animal Technology Co., Ltd. (No. SCXK (zhe) 2019-0001). During the whole experimental period, the mice were raised in the animal room under standard conditions (room temperature 23 ± 2 °C, relative humidity 50 ± 10%, 12 h light and darkness cycle) for 7 days with free access to basal food and water. Ninety-six healthy mice were then divided into six groups randomly (16 mice per group): Normal control group (the NC group); Alcohol model group (the AM group); A single gavage of the highest-dose EULP group (the SE group); Multiple gavages of the low-dose EULP group (the MEL group); Multiple gavages of the medium-dose EULP group (the MEM group); Multiple gavages of the high-dose EULP group (the MEH group).

**Figure 1:**
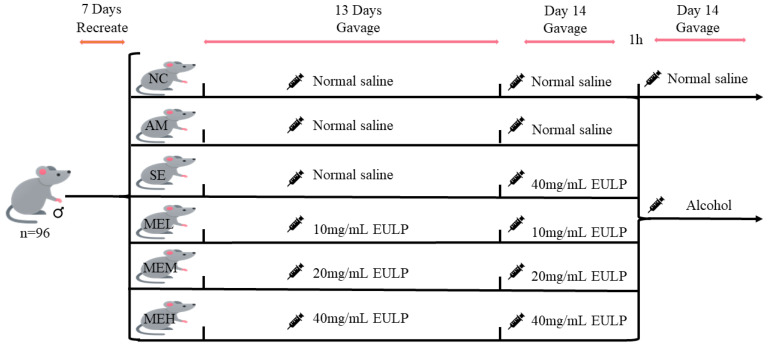
Animal experimental design.

Figure 1 shows the animal experimental design of EULP on mice with acute alcoholism. Briefly, the NC and AM groups received normal saline by gavage, whereas the MEL, MEM, and MEH groups received 10 mg/mL, 20 mg/mL, and 40 mg/mL EULP by gavage under the dose volume of 0.01 mL/g body weight (BW) for 13 days, respectively. On day 14, the first gavage was administered to the AM group and the NC control group with normal saline, while the SE, MEL, MEM, and MEH groups received 40, 10, 20, and 40 mg/mL of EULP, respectively, at a volume of 0.01 mL/g·BW. The second gavage was carried out 1 h later. The AM group and all EULP groups were gavaged with 15 mL/kg·BW of Red Star Erguotou liquor with 56% alcohol (Beijing Red Star, Beijing, China), while the NC group received normal saline of the same volume. After the operation, blood was sampled by eyeball blood sampling. Mice were then slaughtered, and cecum, colon, ileum, cecum contents, ileum contents, and liver tissue were taken quickly. The study was approved by the animal experiment ethics committee of Nanchang University, Nanchang, China. 

### 2.4. Observation of Mice

The loss of the righting reflex was used to judge the drunkenness of mice. All mice were turned downward 5 min after intragastric administration to test whether they were inebriated by waiting until the righting reflex vanished. Mice were classified as inebriated, and their sober and intoxicated time was recorded if they could hold their backward posture for more than 30 s without their righting reflex returning. 

### 2.5. Histopathological Examination

Extracted liver tissues were preserved in a paraformaldehyde solution for 24 h, embedded in paraffin, and then sliced into slices of 4 mm thick. Following hematoxylin and eosin (H&E) staining, the tissue slices were examined using the Aperio LV1 real-time digital pathological scanning system (Leica Biosystems, Wetzlar, Germany), which has a 200-fold field of view.

The liver cell lesions were observed under an optical microscope to evaluate the mouse model of alcoholic liver disease (ALD). The alcoholic liver disease activity score (AAS) standard was established according to the non-alcoholic fatty liver disease activity score (NAS) [32]. AAS score greater than 4 points was diagnosed as ALD, less than 3 points can exclude ALD, 3–4 points was ALD-possible. The specific scoring rules are shown in Table 1.

### 2.6. Serum Biochemistry

After drawing blood from the mice, the blood was allowed to stay at room temperature for 1 h. The static blood was centrifuged at 2000 rpm for 15 min, and the supernatant was collected. Plasma levels of alanine aminotransferase (ALT) and aspartate aminotransferase (AST) were then determined according to respective commercial kits (Nanjing Jiancheng Bioengineering Institute, Nanjing, China). Plasma LPS quantification was performed using a mouse lipopolysaccharide/endotoxin ELISA kit (Nanjing Jiancheng Bioengineering Institute, Nanjing, China), according to the manufacturer’s instructions.

### 2.7. Inflammatory Cytokines and Antioxidant Parameters Determination

An amount of 20 mg of liver or colon tissues were obtained from separate groups of mice, and the homogenate was placed in an ice box and ice-bathed at −20 °C for 4 h after the addition of 200 μL of lysis solution and indirect homogenization for 10 min. After being thoroughly lysed and centrifuged at 10,000 rpm for 10 min, the supernatant was collected. The contents of TNF-α, IL-6, and IL-10 were determined by corresponding enzyme-linked immune sorbent assay (ELISA) kits in accordance with the manufacturer’s protocols (Boster Biological Technology Company, Hubei, China). Glutathione (GSH), superoxide dismutase (SOD), and total antioxidant capacity (T-AOC) were determined by colorimetric method according to respective commercial kits (Nanjing Jiancheng Bioengineering Institute, Nanjing, China). 

### 2.8. Western Blot Analysis

An amount of 20 mg of ileum tissue from mice of each group were extracted, mixed at a 1:10 (mg:L) ratio with prepared Western and IP cell lysates with 1% protease inhibitor (PMSF), and incubated on ice. The uniform tissue homogenates were centrifuged at 12,000 rpm/min for 10 min after full rupture treatment on the tissue homogenizer, and the supernatants were collected and stored at −80 °C until analysis. The protein concentration was measured via bicinchoninic acid assay (BCA) using the BCA protein concentration assay kit (Beyotime Biotechnology, Shanghai, China).

Extracted proteins were separated using SDS-PAGE according to the ExpressCast PAGE color gel kit (Suzhou Xinsaimei Biotechnology company, Suzhou, China) protocol, and then transferred to the PMSF membranes (100 mM). After blocking with a blocking solution, the membranes were incubated with primary antibody (1:1000, diluted with TBST) overnight at 4 °C. After washing 5–6 times with TBST, the membranes were incubated with the corresponding secondary antibodies (1:10,000, diluted with TBST for 1 h. According to the ECLplus kit (Boster Biological Technology Company, Wuhan, China) instructions, protein bands were visualized using the Bio-Rad ChemiDoc Imaging system (Bio-Rad Laboratories, Hercules, CA, USA).

### 2.9. 16S rRNA Gene Amplicon Sequencing of Microbiota

The extraction of DNA from the colon contents of mice was based on the Mag-Bind DNA Kit (Omega Bio-Tek Company, Guangzhou, China). Agarose gel electrophoresis and TAE were used to evaluate the concentration and purity of DNA. The V3 and V4 regions of 16S rRNA were amplified by PCR using the ACTCCTACGGGAGGCAGCA sequence as the upstream primer and the GGACTACHVGGGTWTCTAAT sequence as the downstream primer. After the quality and concentration of the library were qualified, the Illumina platform (Personal Biotechnology Company, Shanghai, China) was used to perform paired-end sequencing of the community DNA fragments [33].

The GenesCloud tool, which is a free online platform for data analysis (https://www.genescloud.cn), was used for analysis and mapping. By using Vsearch (v2.13.4, Linux) and Cutadapt software (v2.3), the Vsearch method based on OTU clustering was used for data analysis, mainly including steps such as primer removal, splicing, quality filtering, deduplication, dechimerization, and clustering. The QIIME2 (2019.4) was used to analyze the alpha diversity and beta diversity index of microorganisms. In addition, we used QIIME2 (2019.4) to obtain the specific composition table of the microbial community in each sample at each classification level by counting the flattened OTU table. Then, the composition distribution of each sample at six classification levels was visualized by counting the feature table after singleton removal, and the analysis results were presented in a histogram.

### 2.10. Statistical Analysis

Statistical analysis was performed by using the statistical analysis software IBM SPSS 19.0. ANOVA analysis and multiple comparison tests were applied to evaluate the differences between different groups. At least three parallel samples were analyzed, and the data were expressed as the mean ± standard deviation (S.D.).

## 3. Results

### 3.1. Characterization of EULP

Brown powdery polysaccharides were prepared from *Eucommia ulmoides* Oliv. leaves via pretreatment, extraction, and preliminary purification. It appeared as a heterogeneous mixture of polysaccharides consisting of both 75% sugars and 1.64% protein (Table 2). The molecular weights, number average molecular weight (Mn), and Z average molecular weight (Mz) of EULP were calculated to be 186,132, 82,991, and 345,033 Da, respectively.

**Figure 2:**
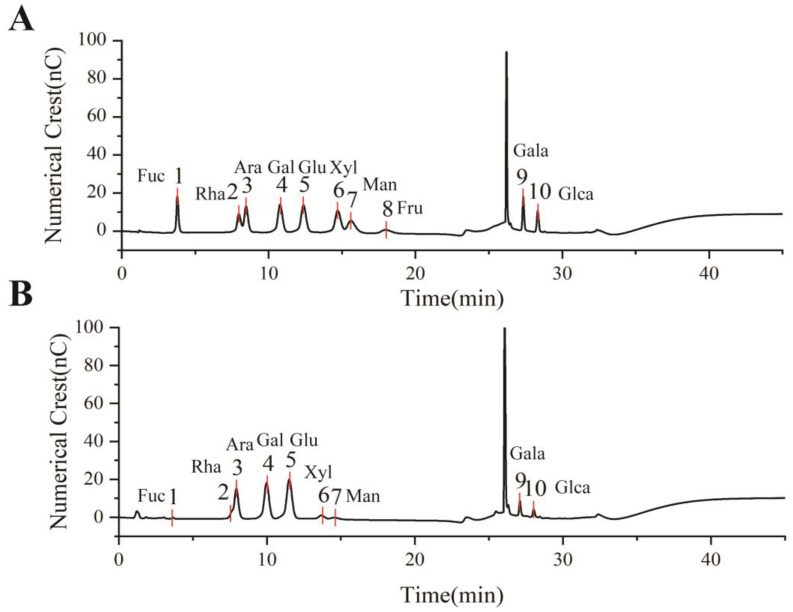
Monosaccharide analysis of EULP using an ion chromatogram. (**A**) The retention time of 10 monosaccharide standards. (**B**) The ion chromatogram of EULP.

The monosaccharide composition of EULP was analyzed through an ion chromatogram, and the retention times were compared with those of monosaccharide standards. As shown in Figure 2, EULP mainly contains fucose, rhamnose, arabinose, galactose, glucose, xylose, galactose acid, and glucuronic acid. According to Table 2, the molar proportions of fucose, glucuronic acid, and mannose were less than 3%, which might vary considerably in different samples or extracting methods. The most abundant sugar was glucose, followed by galactose, arabinose, and galactose acid.

### 3.2. Animal Observation

Because higher doses of alcohol create central depressive effects, causing sleep behavior in mice, loss of the righting reflex (LORR) is applied as one of the markers for determining whether mice have been poisoned by alcohol. In this study, all groups of mice were in good condition, responsive, and energetic during the 13 days of EULP or normal saline gavage. Then, 30 min after intragastric administration of alcohol, mice showed acute alcoholism such as mental malaise, urinary incontinence after alcohol, dyspnea, and LORR. Mice in the AM group did not perish, and the duration of LORR in mice of the NC group was shorter than that of the AM group, suggesting successful establishment of the model. Next, 220 min after alcohol administration, mice in the AM group persisted in an unconscious state, whereas mice in the EULP groups woke up one after another.

### 3.3. EULP Ameliorates Pathological Characteristics of Hepatic Damage in Acute Alcoholic Mice

**Figure 3:**
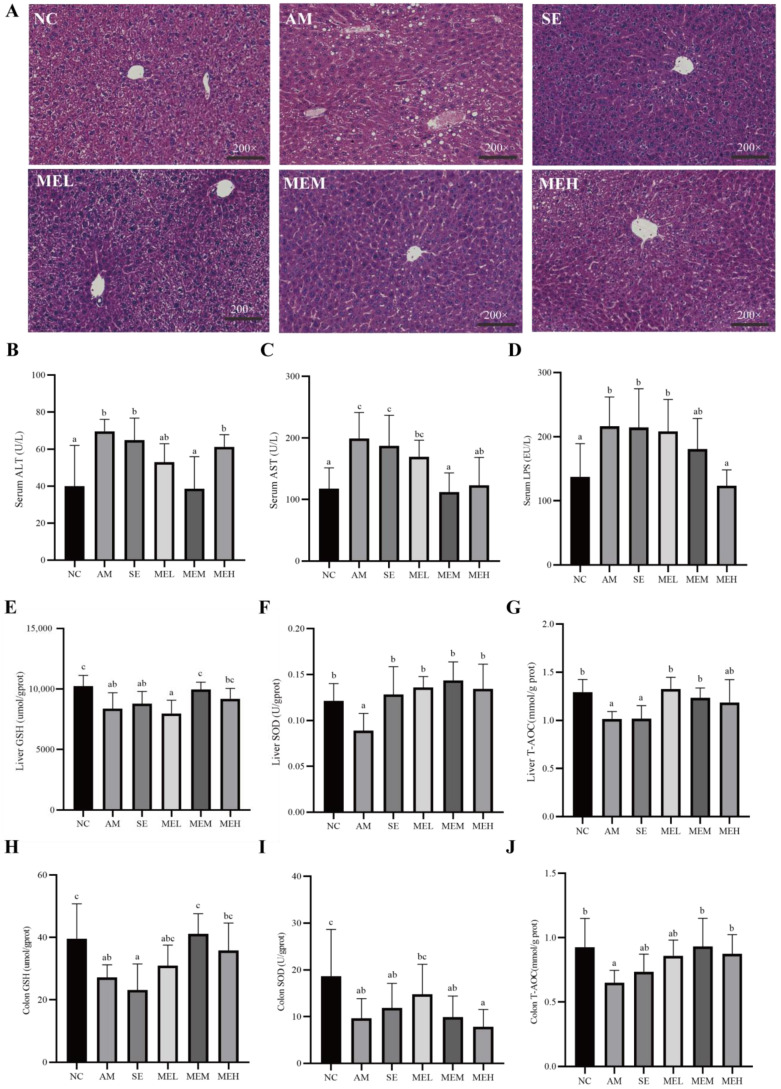
EULP alleviated liver and intestine injury and oxidative stress in mice with acute alcoholism. (**A**) Representative microscopic images of mouse liver after H&E staining (200×). Quantification of ALT (**B**) and AST (**C**) in mice serum. (**D**) Quantification of LPS in mice serum. Quantification of GSH (**E**), SOD (**F**), and T-AOC (**G**) in mice liver. Quantification of GSH (**H**), SOD (**I**), and T-AOC (**J**) in mice colon. Data are presented as mean ± S.D. (*n* = 6) and analyzed using one-way ANOVA followed by Tukey’s post hoc test. Groups with different letters are significantly different from each other.

Pathological observations revealed that the liver cells were arranged closely and orderly with uniform size and obvious cell boundaries in the NC group. Differently, in the AM group, hepatocytes were arranged in disorder, with cytosolic edema, vacuolation, and fatty accumulation. However, both 1-day and 14-day pretreatment with EULP alleviated acute alcohol-induced morphological changes. Specifically, hepatocytes from mice in these treatment groups revealed reduced cell necrosis and significantly better fat vacuole status, and the morphology of medium and high doses of EULP administration was similar to that of the NC group (Figure 3A). The AAS of ALD mice are shown in Table 3, further demonstrating the impact of EULP on alcohol-induced liver injury in mice.

High blood enzymatic activities of ALT and AST are commonly employed as indications of liver injury, which could be attributed to the release of these cytoplasmic components into the systemic circulation following hepatic cell destruction [34]. As shown in Figure 3B,C, the levels of plasma ALT and AST activities were markedly elevated in mice from the AM group in comparison to those from the NC group. However, 14 days of gavage EULP administration significantly alleviated plasma ALT and AST levels in mice compared to changes in the AM groups. For the reason that elevated LPS levels have been noted as an aggravating factor in alcoholic liver injury, the concentration of serum LPS was determined. As shown in Figure 3D, the level of serum LPS was significantly higher in the AM group than in the NC group, which was reversed by a 14-day pretreatment of a high dose of EULP.

### 3.4. EULP Improves Hepatic and Colonic Antioxidant Status in Acute Alcoholic Mice

As tissue damage is always coupled with abnormal oxidative stress which can be alleviated by antioxidants, several indicators for the antioxidant status of tissues including non-enzymic antioxidants (GSH and T-AOC) and enzymic antioxidant (SOD) were evaluated [35,36]. As shown in Figure 3E–G, the activities of all antioxidants in the liver of the acute alcohol-induced mice were significantly decreased compared to that of the NC group. The 14-day pretreatment with EULP at various doses significantly increased the hepatic GSH, T-AOC, and SOD content as compared to the AM group, which return to normal levels. For short-time EULP pretreatment, there was a significant increment in the SOD level, but hepatic non-enzymic antioxidants showed no significant difference compared to the AM group.

Similar impacts of EULP on the levels of GSH, SOD, and T-AOC in the colon tissue of alcohol-exposed mice are shown in Figure 3H–J. Specifically, activities of GSH, T-AOC, and SOD were remarkably downregulated in the AM group in comparison to the NC group. The 14-day pretreatment with EULP at various doses significantly increased the activities of GSH and T-AOC in the colon tissue of mice in comparison to in the AM group and tended to be normal; however, the SOD level in the colon tissue of mice showed no significant difference.

### 3.5. EULP Ameliorates Hepatic Inflammation in Acute Alcoholic Mice

**Figure 4:**
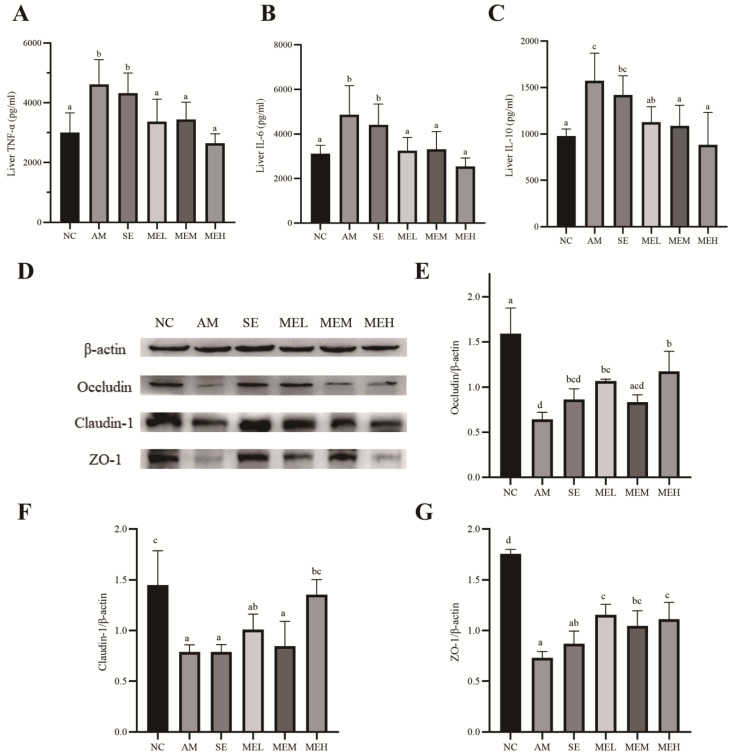
EULP alleviated liver inflammation and colon TJ protein expression in mice with acute alcoholism. Quantification of TNF-α (**A**), IL-6 (**B**), and IL-10 (**C**) in mice liver (*n* = 6). Western blotting for the tight-junction proteins (TJ) in the colon (**D**). Quantitative analysis of Occludin (**E**), Claudin-1 (**F**), and ZO-1 (**G**) in the colon tissue (*n* = 3). Data are presented as mean ± S.D. (*n* = 6) and analyzed using one-way ANOVA followed by Tukey’s post hoc test. Groups with different letters are significantly different from each other.

We assessed the effects of EULP on the inflammatory responses in the liver by measuring the expression levels of proinflammatory cytokines (TNF-α and IL-6) and regulatory cytokine IL-10. As shown in Figure 4A–C, alcohol administration significantly elevated liver expression of TNF-α, IL-6, and IL-10 in comparison to the NC group. However, unlike short pretreatment (the SE group), 14 days of EULP pretreatment significantly lowered the alcohol-induced increase in cytokines in the liver, almost restoring them to normal levels.

### 3.6. EULP Reduces Intestinal Hyperpermeability in Acute Alcoholic Mice

Previous studies demonstrated that acute alcohol exposure influenced the formation of tight junctions (TJ), which is critical for maintaining intestinal integrity [37,38]. In this study, the effect of EULP on TJ proteins Occludin, Claudin-1, and ZO-1 in the colon of mice was detected by Western blot. As shown in Figure 4D–G, after alcohol exposure, the expressions of Occludin, Claudin-1, and ZO-1 protein in the AM group were significantly lower than those in the NC group. Fourteen days of EULP pretreatment significantly increased Occludin, Claudin-1, and ZO-1 levels to a different extent.

### 3.7. EULP Prevents Intestinal Flora Disorder in Acute Alcoholic Mice

#### 3.7.1. Sequence Processing Analysis Results, OTU Analysis, and Species Composition Analysis

A total of 18 samples were measured, with 6 of them in the NC, AM, and MEH groups, and a total of 1,731,194 sequences were obtained. The high-quality sequences were clustered at the 97% similarity level, and the representative sequences and OTU tables were output respectively.

Through OTU analysis, the number of unique and common OTUs in each group can be shown. The unique OTU numbers of the NC group, AM group, and the MEH were 961, 1460, and 1753, respectively. The number of OTUs in the AM group, and the MEH and NC groups, was 151 and 201, respectively, indicating that the OTU similarity between the MEH group and the normal group was higher than that of the model group. The total number of OTUs was 611. The average OTU of each sample was 682.8333. The results from the OTU analysis indicated that the rank–abundance curve of bacterial communities in the NC, AM, and MEH groups exhibited a similar pattern, albeit with different microbial compositions.

The number of species of each sample were counted and explored using SPSS23.0 at different taxon levels. The abundance of phylum, class, order, family, genus, and species in all samples detected was high, with 9.83, 14.78, 20.78, 37.22, 89.78, and 34.83 taxa, respectively.

#### 3.7.2. Alpha Diversity and Beta Diversity Analysis

**Figure 5:**
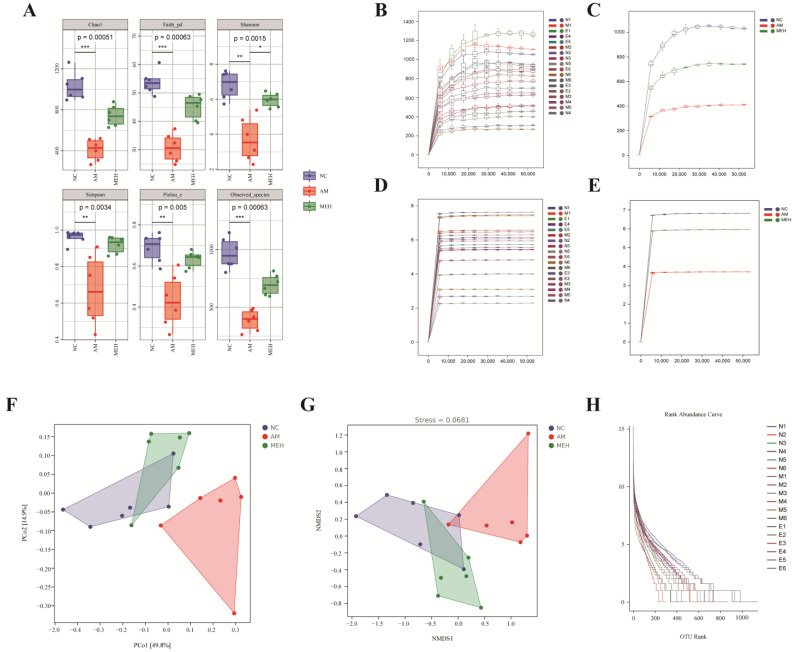
Alpha diversity and beta diversity analysis. (**A**) Chao, Faith-pd, Shannon, Simpson pielou-e, and observed species were determined to assess the α-diversity of gut microbiota; (**B**) The dilution curve of a single sample; (**C**) The dilution curve of each group of samples; (**D**) The Shannon dilution curve of a single samples; (**E**) The Shannon dilution curve of each group created by Shannon algorithm; (**F**) The PCoA analysis of intestinal flora; (**G**) The NMDS analysis diagram; (**H**) The rank–abundance curve of OTUs in each sample. Data were reported as mean ± S.D. Statistical analysis was calculated by using the independent Student-*t* test. Asterisk * indicated *p* < 0.05; ** indicated *p* < 0.01; *** indicated *p* < 0.001.

To comprehensively evaluate the alpha diversity of microbial communities, a serious of indices were applied. As shown in Figure 5A, compared with the NC group, the Shannon index of the AM group was significantly reduced, and the Shannon index was significantly increased after the treatment of EULP. It showed that the diversity of intestinal flora in mice after alcohol treatment will be affected, and EULP can alleviate this effect.

The beta diversity index is more inclined to compare the differences between the samples, thus reflecting the different community differences between the two samples. Figure 5F was obtained by distance matrix and PCoA analysis. PCo1 and PCo2 accounted for 49.8% and 14.9% of the overall analysis data, respectively. Compared with the NC group and the MEH group, the AM group showed obvious separation. The results showed that EULP had a certain regulatory effect on the disorder of intestinal flora caused by alcohol. Non-metric multidimensional scaling analysis (NMDS) was similar to PCoA analysis, unaffected by the value of sample distance, and only evaluates the size relationship between them. For data with complex structure, the ranking result may be more stable. As shown in Figure 5G, the stress value (Stress) of NMDS results was 0.0681, and the results of NMDS analysis were more reliable. At the same time, there was a significant separation between the AM group and the NC group and the MEH group, indicating that alcohol could cause intestinal flora disorder in mice, and EULP could alleviate this disorder. The abundance curve in Figure 5H shows that the broken line was gradually gentle, indicating that the uniformity of community composition was good, and the abundance difference between OTUs in the community was small.

#### 3.7.3. Species Composition Analysis

**Figure 6:**
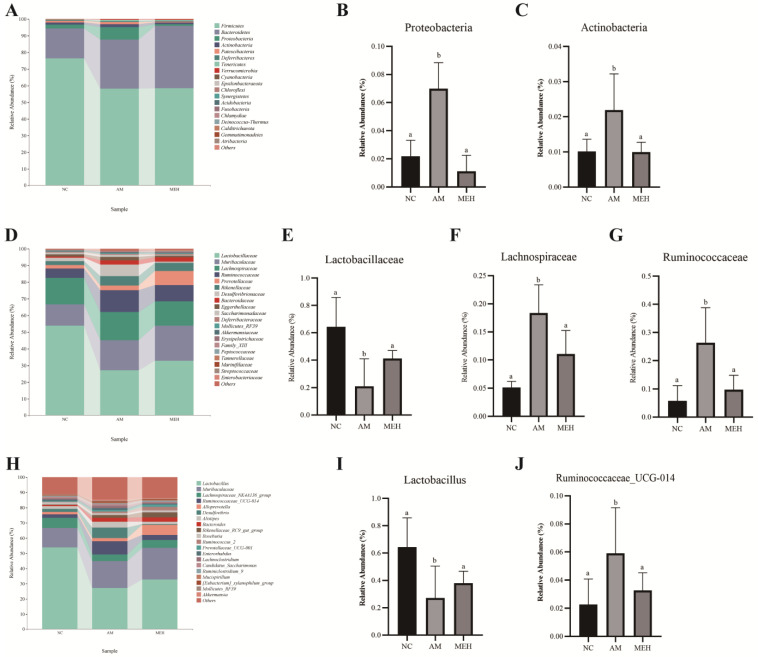
Species composition analysis. (**A**) Histogram of species distribution at the phylum level; (**B**) The relative abundance map of *Actinobacteria*; (**C**) The relative abundance map of *Actinobacteria*; (**D**) Family-level species distribution histogram; (**E**) Relative abundance of *Lactobacillus* family; (**F**) The relative abundance map of *Lachnospiraceae*; (**G**) The relative abundance map of *Verrucomicrobia*; (**H**) A bar chart of species distribution at the genus level; (**I**) The relative abundance map of *Lactobacillus*; (**J**) The relative abundance map of *Ruminococcaceae* UCG-014. Significance was determined by one-way ANOVA followed by Tukey’s analysis (*p* < 0.05). Labeled characters without a common letter represent significant differences from the other group(s).

As shown in Figure 6A–C, the phylum Firmicutes, Bacteroidetes, Proteobacteria and Actinobacteria were the most abundant bacterial group in the colon contents of mice. Compared to the NC group, the relative abundance of Proteobacteria and Actinobacteria in the AM group increased significantly, whereas decreased significantly after 14 days of pretreatment of EULP.

At the family level, *Lactobacillaceae*, *Muribaculaceae*, *Lachnospiraceae*, *Ruminococcaceae*, *Prevotellaceae*, and *Rikenellaceae* had high relative abundance, as shown in Figure 6D–G. Compared to the NC group, the relative abundance of *Lactobacillaceae* in the AM group significantly decreased, while *Lachnospiraceae* and *Verrucomicrobiaceae* increased significantly. However, 14-day EULP pretreatment showed an opposite trend.

At the genus level, the high relative abundance of *Lactobacillus*, *Muribaculaceae*, *Lachnospiraceae* NK4A136 group and *Ruminococcaceae* UCG-014 group was in descending order, as shown in Figure 6H–J. In comparison with the NC group, the AM group exhibited a notable decrease in the relative abundance of *Lactobacillus* and an increase in the relative abundance of *Ruminococcaceae* group UCG-014. The 14-day EULP pretreatment induced an increase in *Lactobacillus* abundance and a reduction in *Ruminococcaceae* group UCG-014 abundance, although with no significance.

#### 3.7.4. Species Differences and Marker Analysis

**Figure 7:**
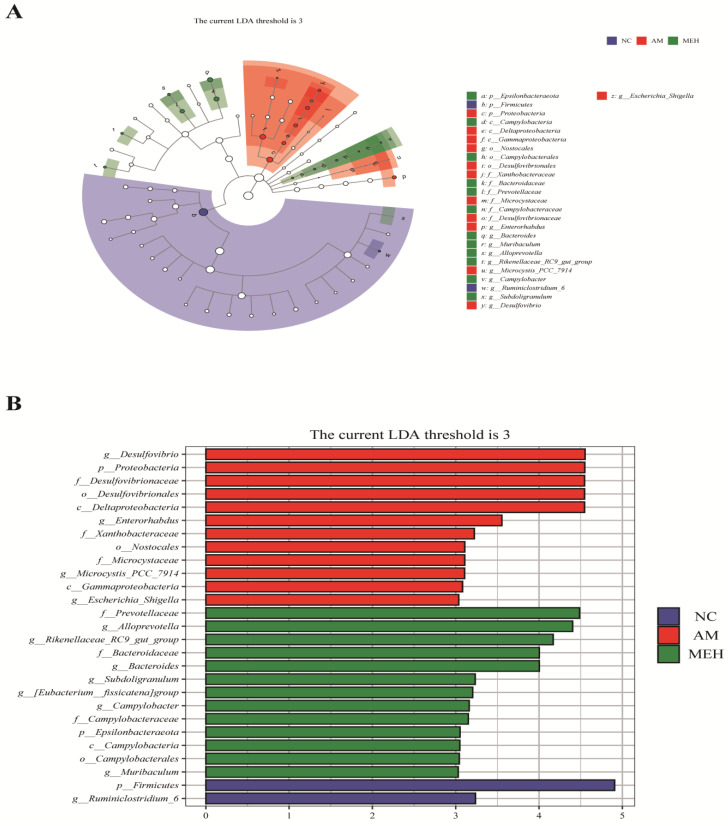
LEfSe analysis of intestinal flora in colon. (**A**) LDA value distribution histogram of significantly different species; (**B**) The Taxonomic branch diagram.

LEfSe (LDA Effect Size) analysis is a frequently utilized method for difference analysis, which can not only directly compare all classification levels at the same time but also identify robust and reliable potential biomarkers. Twenty-six biomarkers were screened by LEfSe analysis in this study. According to Figure 7, 27 different levels of taxa were identified with varying degrees of richness, with the AM group having the largest number of differentiated groups, Specifically, *p-Firmicutes* was more prevalent in the NC group, species *g-Desulfovibrio* was overrepresented in the AM group, and strain *f-Prevotellaceae* was predominant in the EULP group. Previous literature showed that the *g-Desulfovibrio* genus is harmful to the human body [39], but *f-Prevotellaceae* is an intestinal beneficial bacterium [40]. Notably, EULP pretreatment increased the beneficial bacteria in the intestinal flora of mice affected by alcohol. These results indicated that EULP pretreatment could restore acute alcohol-induced disorder of intestinal flora, through promoting the abundance of beneficial bacteria and reducing the relative abundance of harmful bacteria.

### 3.8. Correlation Analysis between Intestinal Microflora and Liver Injury Markers

**Figure 8:**
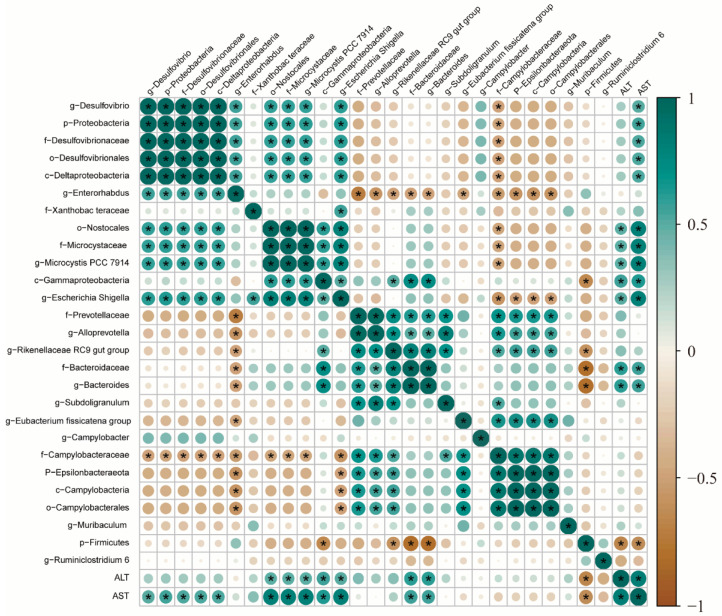
Correlation analysis between the relative abundance of the main genus in the gut microbiota and the liver injury biomarkers (ALT and AST). Blue squares represent positive correlations, brown squares represent negative correlations, and white squares represent no correlations. * indicates a significant *p* value < 0.01.

We then analyzed the relationship between liver injury markers and intestinal microbes. Pearson analysis was used to analyze the correlation between intestinal flora and liver injury markers. Figure 8 illustrates the correlation test results of the Pearson algorithm calculation, *c-Gammaproteobacteria* at the class level, *o-Nostocales* at the order level, *f-Microcystaceae* and *f-Bacteroidaceae* at the family level, and *g-Microcystaceae* PCC-7914, *g-Escherichia-Shigella*, and *g-Bacteroides* at the genus level were positively associated with ALT level, whereas *g-Muribaculum* at the genus level had a negative relationship with ALT level. Similarly, *p-Proteobacteria* at the phylum level, *c-Gammaproteobacteria*, *c-Deltaproteobacteria*, and *c-Deltaproteobacteria* at the level of class, *o-Nostocales*, *o-Desulfovibrionales*; the *f-Microcystaceae*, *f-Bacteroidaceae*, and *f-Desulfovibrionaceae* at the family level and the *g-Microcystis* PCC-7914, *g-Escherichia-Shigella*, *g-Bacteroides*, and *g-Desulfovibrio* at the genus level were positively correlated with AST level, whereas *g-Muribaculum* at the genus level had a negative relationship with AST level.

## 4. Discussion

As the primary organ of alcohol metabolism, various degrees of liver impairment may occur with excessive drinking. Hepatocytes that are injured by alcohol undergo cloudy swelling, increase cell permeability, and result in the release of cytosolic marker enzymes ALT and AST, which are mostly found in hepatocyte cytoplasm and hepatocyte mitochondria, respectively, into the blood. Hence, the activities of ALT and AST in serum are the most intuitive biochemical parameters reflecting the degree of liver damage [41,42,43]. In this study, excessive intake of alcohol remarkably increased serum levels of both ALT and AST, indicating that hepatocyte membrane damage reached organelle level. Long-term but not short-term EULP pretreatment has a good effect on decreasing ALT and AST activities, indicating that EULP could protect liver tissue from alcohol-induced degradation. The histopathological observation also confirmed the above view.

There are convincing data that acute ethanol treatment enhances oxidative stress, contributing to liver injury [44]. We here investigated the antioxidant defense system of alcohol-induced liver injury mice. Alcohol abuse decreased the levels of SOD, GSH, and T-AOC, which was consistent with previous reports. Long-term early EULP administration increased the body’s ability to deal with oxidative damage. It was noteworthy that there was no therapeutic effect of short-term EULP pretreatment except for the activity of SOD.

The resident liver macrophages Kupffer cells are often involved in acute alcoholic liver injury. They are activated by endotoxin and produce the pro-inflammation cytokine TNF-α, the hepatoprotective cytokine IL-6, and the anti-inflammatory cytokine IL-10. In this study, a single intoxicating dose of alcohol induced massive induction of TNF-α in the liver tissue, which was not offset by the increase in IL-10 expression. EULP pretreatment has a good effect on decreasing cytokine concentrations, which may be related to a reduced blood level of LPS from the intestine. The therapeutic effect of long-term EULP pretreatment in decreasing cytokine concentrations is dose-dependent and better than that of short-term EULP pretreatment. In addition, the amounts of cytokines in serum did not alter significantly, demonstrating that acute alcohol does not cause systemic inflammation. These results prompted the concept that the protective effects of EULP on acute alcoholism mice may have an intestinal-based mechanism.

Because the intestine absorbs 70% of alcohol and transports it through the portal venous system to the liver, where it is processed, the intestine can have a significant impact on the liver via a variety of by-products and substances, which is often referred to as the gut–liver axis [45]. Alcohol-induced disorder of gut microbiota and alteration of their by-products superimposed on a background of intestinal barrier dysfunction may lead to the injury of the liver [46,47]. On one hand, intestinal permeability, which is a feature of intestinal barrier function, was proven to be elevated by a single oral dose of alcohol. TJ proteins between intestinal epithelial cells, including transmembrane protein Occludin and Claudin-1, interact with zonula occludens proteins and control the passage of molecules through the paracellular space [48]. It has been reported that natural polysaccharides can effectively repair the alcohol-injured intestinal barrier and maintain the integrity of barrier function by increasing the expression of tight junction proteins [49]. Similarly, we found that the improvement of EULP on the intestinal mucosal barrier was reflected by increasing Occludin, Claudin-1, ZO-1 levels, and decreasing serum LPS level, which formed from intestinal dysbiosis and contribute to the activation of resident liver macrophages Kupffer cells involving in alcoholic liver injury.

On the other hand, excessive alcohol consumption significantly alters the gut microbiota and microbiota-derived metabolites, thus destroying the intestinal barrier. In this work, the results of 16S rRNA gene sequencing further verified that alcohol exposure leads to the ecological imbalance of the microflora, the increase in rare microflora, and the change in microbial composition at various classification levels. However, EULP greatly reduced these changes almost at all taxonomic levels including *Proteobacteria*, *Actinobacteria*, *Lachnospiraceae*, and *Verrucomicrobia*, which all returned to normal levels. A large number of studies have also shown that liver diseases were associated with altered microbial diversity in the intestines, which may in turn exacerbate liver injury [50,51]. Patients with alcoholic liver illness generally contracted *Bacteroides* species and enlarged *Proteobacteria* species. Additionally, dysbiosis of the intestinal flora is also associated with a marked elevation of serum endotoxin due to excessive bacterial translocation. The presence of endotoxemia and reduced abundance of *Bacteroides* negatively affects liver regeneration [52]. Other etiologies, such as digestive tract diseases, can also influence liver damage by regulating gut microbes. Supplementation with *Lactobacillus casei* and *Bifidobacterium lactis* probiotics is able to ameliorate the inflammation and apoptosis in both the colon and liver caused by TNBS in mice. Compared to the results of previous studies, we found that AST was positively correlated with *g-Desulfovibrio* and negatively correlated with *g-Muribaculum*. *G-Desulfovibrio* is an opportunistic pathobiont and is associated with liver cirrhosis [53]. Together, hepatic metabolism, microbiome, and liver injury all have a close relationship and should be researched further.

## 5. Conclusions

Our results showed that 14-day rather than single EULP pretreatment can effectively prevent alcohol-induced liver damage, which can be demonstrated by reducing serum marker levels (ALT and AST), alleviating liver inflammation cytokines (TNF-α, IL-6, and IL-10), and significantly restoring liver and colonic antioxidants (SOD, GSH, and T-AOC) and intestinal barrier, as evidenced by the stability of intestinal microecology, reduced intestinal hyperpermeability, and endotoxin infiltration. In summary, EULP exhibited positive effects on acute alcohol-induced hepatic oxidative stress, inflammation, and liver damage through the microbiota–gut–liver axis in mice. These associative findings suggested that the active ingredient EULP in *E. ulmoides* Oliv. leaves is a beneficial compound to treat acute alcohol-exposure-induced liver injury and microbiomic disturbance.

## Figures and Tables

**Table 1:** Alcoholic liver disease activity score standard.

Project	Degree	Score
Hepatic steatosis	<5%	0
	5–33%	1
	34–66%	2
	>66%	3
Intralobular inflammation	0	0
	<2	1
	2–4	2
	>4	3
Hepatocyte ballooning	No	0
	Rare	1
	Common	2

**Table 2:** Monosaccharide composition of EULP. The types and corresponding contents of monosaccharide in EULP are shown.

	Monosaccharide	Molar Percent (%)
1	Fucose	0.48
2	Rhamnose	4.42
3	Arabinose	18.69
4	Galactose	29.46
5	Glucose	35.90
6	Xylose	3.19
7	Mannose	1.50
8	Fructose	0.00
9	Galactose acid	3.82
10	Glucuronic acid	2.52

**Table 3:** Liver pathological score of ALD mice. Data are presented as mean ± S.D. (*n* = 6) and analyzed using one-way ANOVA followed by Tukey’s post hoc test. Groups with different letters are significantly different from each other.

Groups	AAS
NC	0.72 ± 0.29 ^a^
AM	1.29 ± 0.35 ^b^
SE	1.21 ± 0.63 ^b^
MEL	0.83 ± 0.51 ^ab^
MEZ	0.86 ± 0.27 ^ab^
MEH	0.70 ± 0.30 ^a^

## Data Availability

The original contributions presented in the study are included in the article/Supplementary Materials, further inquiries can be directed to the corresponding author.

## Supplementary Materials

The following supporting information can be downloaded at: https://www.mdpi.com/article/10.3390/foods13071089/s1, Figure S1. UV spectra of EULP (A), FTIR spectra of EULP(B); Figure S2. X-ray Diffractogram of EULP(A). Thermal analysis diagram of EULP (B). Differential scanning calorimetry (C). SEM images of EULP magnification 100× (D). SEM images of EULP magnification 500×(E). SEM images of EULP magnification 10000×(F); Figure S3. Size distribution of EULP(A). Zeta potential of EULP(B).
