# Supplementary material for: Polysaccharides from Eucommia ulmoides Oliv. Leaves Alleviate Acute Alcoholic Liver Injury by Modulating the Microbiota–Gut–Liver Axis in Mice
Source: Foods. 2024 Apr 2;13(7):1089. doi: 10.3390/foods13071089 (PMC11011369; doi:10.3390/foods13071089)
Supplement: Supplementary file 1 [file foods-13-01089-s001.zip › foods-2934061-supplementary.pdf]

1.Physicochemical properties of EULP

1.1 Extraction and UV analysis

Brown powdery polysaccharides were derived via pretreatment, extraction and preliminary purification, with a yield of 1.69% based on dry matter. It appeared as a heterogeneous mixture of polysaccharides which consisted of both sugars (75%) and protein (1.64%). As shown in Figure S1A, the full-wave ultraviolet absorption spectrum was used to detect the presence of nucleic acids and proteins in polysaccharides. The weak absorption of EULP solution at the wavelength of 260-280 nm indicated that a small amount of nucleic acid and protein was present, consistent with results determined by the Coomassie Brilliant Blue method. These preliminary results indicated that fair purity of EULP could still be achieved despite the lack of additional purification employing cellulose column chromatography.

1.2 FT-IR characterization

Fourier transform infrared (FT-IR) spectroscopy was performed in the wavelength range of 4000-400 cm^{-1} to further characterize and corroborate the data so far obtained for the polysaccharides present in EULP. As shown in Figure S1B, the absorption peak at 3417 cm^{-1} was ascribed to the stretching vibration of O-H or amino N-H, and the absorption peak at 2926 cm^{-1} was the stretching vibration of C-H. The absorption peak at 2361 cm^{-1} was the stretching vibration of three or two bonds, which were also the characteristic absorption peaks of polysaccharides. Furthermore, the band at 1607 cm^{-1} was due to the C=O carbonyl stretching vibration. The absorbance at approximately 1402 cm^{-1} corresponded to C-H angular variation vibration, while the band at 1000–650 cm^{-1} was unsaturated C-H in-plane bending vibration. The absorption at 1076 cm^{-1} and 1233 cm^{-1} indicated a pyranose unit, and the region around 809 cm^{-1} in EULP was characteristic of β-glycosides.

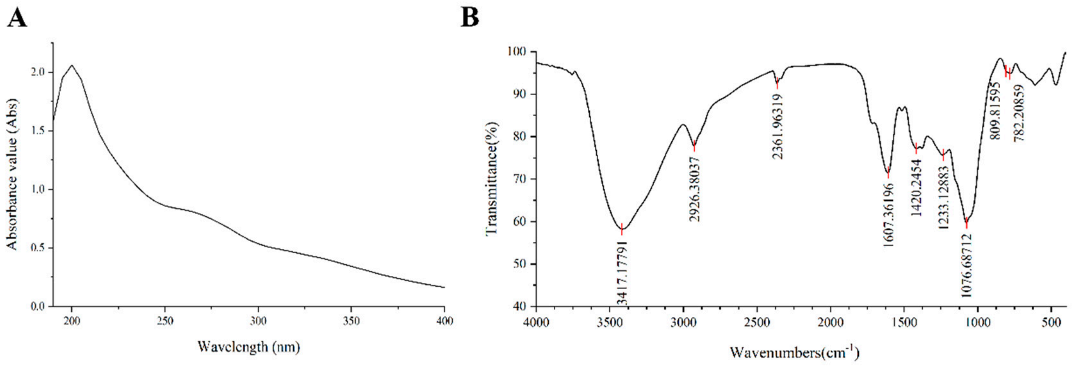

Figure S1. UV spectra of EULP (A), FTIR spectra of EULP(B).

1.3 XRD analysis

In order to investigate the crystallinity of EULP, the XRD pattern of EULP was shown in Figure S2A. The peak structure of a typical amorphous material can be seen in the X-ray spectra of EULP. EULP was a semi-crystalline polymer with major crystalline reflection at 20.49°.

1.4 TG analysis

TG analysis and differential scanning calorimetry (DSC) were used to assess the thermal stability of EULP. According to Figure S2B, the initial mass loss of EULP occurred between 35 and 150°C, with a mass loss of 10.5%. This step was associated with an endothermic peak as shown in the DSC scan (Figure S2C). The evaporation of free water and bound water in EULP could be the primary source of mass loss. In the range of 155-380°C, the weight loss of EULP was 38.2%, and the DSC plot showed a significant endothermic peak. This widespread loss may be due to the thermal decomposition of the polysaccharides. As the pyrolysis temperature increased to 380°C, the mass decreased slowly, the weight loss trend slowed, and the majority of the polysaccharides were carbonized into ash and inorganic components. These results indicated good thermal stability of EULP below 380°C.

1.5 SEM analysis

Figure S2(D-F) revealed the apparent structure of EULP with different magnifications (100×, 500× and 1000×). The microstructure of EULP exhibited an irregular, broken, massive shape. When viewed under high magnification, the surface structure of EULP was rough, with many pores and cracks.

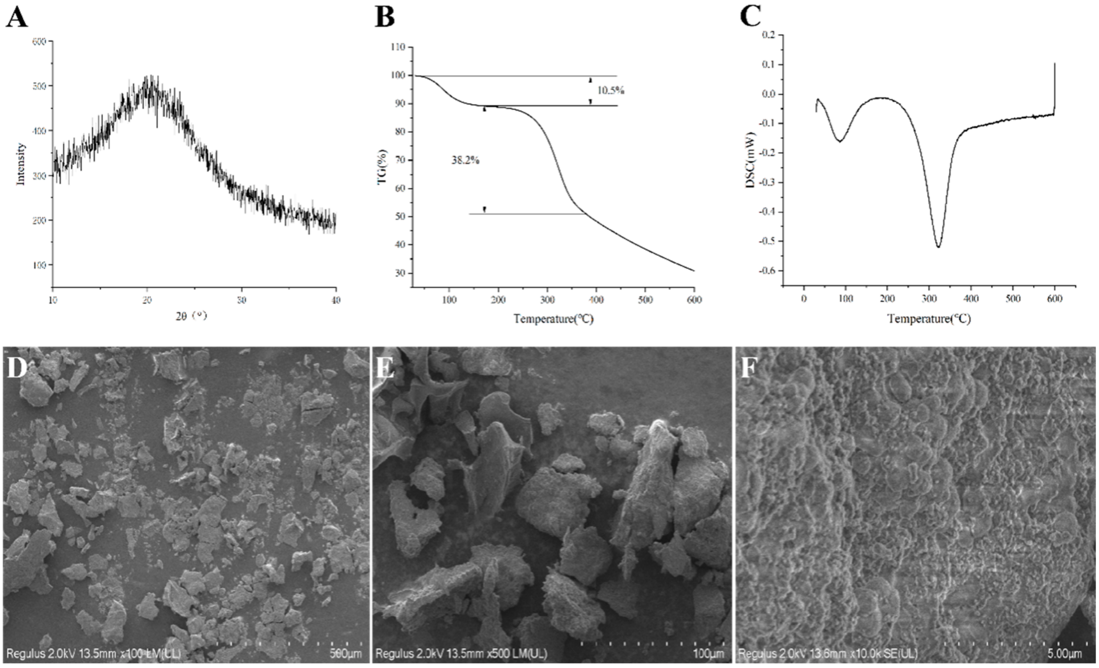

Figure S2. X-ray Diffractogram of EULP(A). Thermal analysis diagram of EULP (B). Differential scanning calorimetry (C). SEM images of EULP magnification 100× (D). SEM images of EULP magnification 500×(E). SEM images of EULP magnification 10000×(F).

1.6 The particle size distribution and Zeta potential

Characterization of hydrodynamic particles and polysaccharide charge can be indicative of the stability of a solution or colloid. Therefore, the particle size and distribution of the EULP fraction were determined. As shown in Figure S3(A), the particle size distribution curves of EULP were two peaked, indicating that the polysaccharide did not form completely uniform droplets, which was consistent with the molecular weight results of EULP determined by GPC. The average particle size was 100.2 nm, and the polydispersity index was 0.235. These results indicated that the average particle size of EULP was small and the uniformity of particle size distribution was general, indicating that EULP may be easily absorbed by the human body.

Zeta potential, which is brought on by the interaction of particles in colloids, can be used as the representation of the stability of the mixed system. In general, the higher absolute value of Zeta potential represented greater electrostatic repulsion between particles and better physical stability. As shown in Figure S3(B), the Zeta potential of EULP solution was 23.4 mV, indicating that EULP had a high dispersion in solution, good stability and was not easily aggregated.

A

B

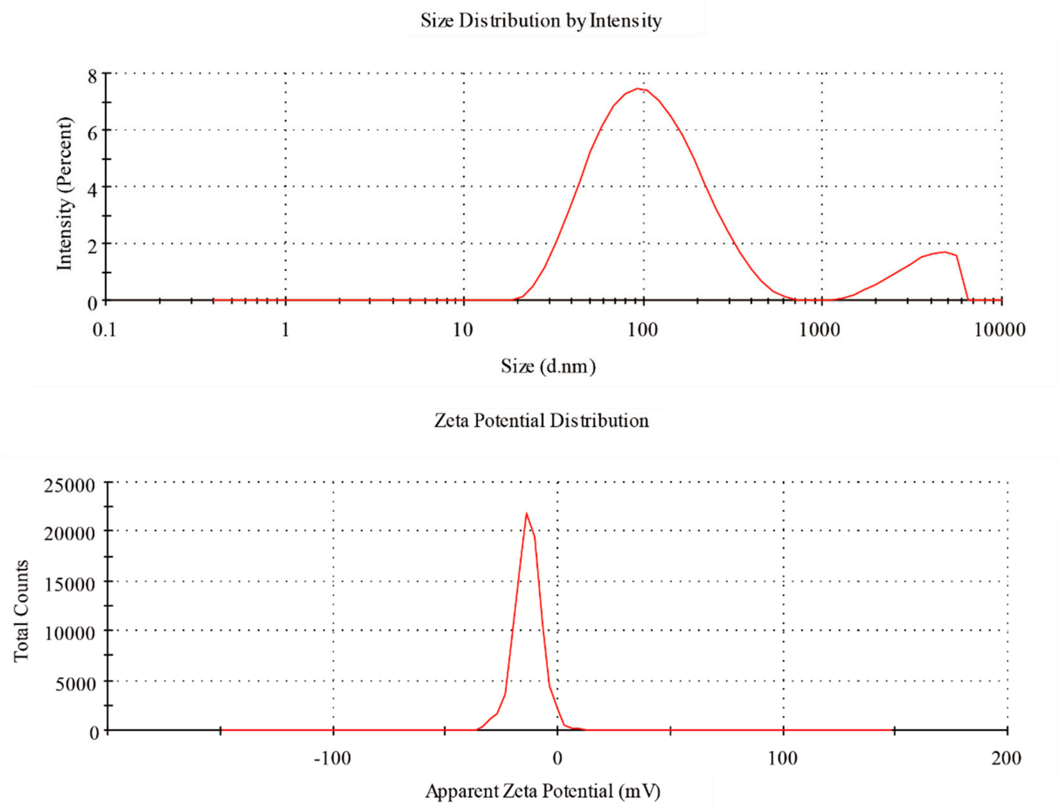

Figure S3. Size distribution of EULP(A). Zeta potential of EULP(B).
